# Maternal Health Workforce Expansion and Local Childbirths

**DOI:** 10.1001/jamanetworkopen.2025.56775

**Published:** 2026-02-02

**Authors:** Yanlei Ma, Olesya Baker, Fang Zhang, Carrie Cochran-McClain, Anjali Kaimal, Hao Yu

**Affiliations:** 1 Department of Health Policy and Management, Harvard T. H. Chan School of Public Health, Boston, Massachusetts; 2 Department of Population Medicine, Harvard Medical School and Harvard Pilgrim Health Care Institute, Boston, Massachusetts; 3 National Rural Health Association, Washington, DC; 4 College of Medicine, University of South Florida, Tampa

## Abstract

This cohort study explores the likelihood of local childbirths in US counties before and after the 2009 expansion of the maternal health clinician workforce via the National Health Service Corps.

## Introduction

Substantial disparities in maternal care access persist across the US.^1,2^ One contributing factor is the maternity care workforce shortage in nearly half of US counties.^3^ The National Health Service Corps (NHSC) supports clinicians to work in Health Professional Shortage Areas (HPSAs) through scholarships and loan repayments.^4^ Since 2009, the NHSC has increased its maternity care workforce in primary care HPSAs by more than 50% (eAppendix in Supplement 1)^5,6^; however, whether this expansion has improved access to local childbirth care remains unknown. We hypothesized that by increasing the number of local clinicians capable of providing prenatal and delivery care, the NHSC expansion could reduce the need for pregnant individuals to seek care outside their home county.

## Methods

The Harvard Pilgrim Health Care Institute Institutional Review Board approved this cohort study as non–human participant research; therefore, informed consent was waived. We followed the STROBE reporting guideline.

We obtained birth certificate data for 2005 to 2019 from the Centers for Disease Control and Prevention and NHSC maternity care clinician information from the Health Resources and Services Administration (HRSA). We classified primary care HPSA counties without any NHSC maternity care clinicians as control counties. Counties without such clinicians before the 2009 NHSC expansion but receiving them afterward were classified as treated counties (eMethods in Supplement 1).

Local births occurred within the maternal county of residence. We used a generalized difference-in-differences design to compare changes in likelihood of local birth between treated and control counties before and after 2009. We used logistic regression to estimate the probability of local birth, with a county-year binary indicator for NHSC maternity care clinician presence, adjusting for individual-, county-, and state-level factors, and fixed effects for county and year-month. To assess effect heterogeneity, we stratified analyses by maternal demographics (self-reported race and ethnicity, education level, and preexisting health risks) and county characteristics (Social Vulnerability Index [SVI], rurality [urban vs rural], receiving NHSC clinicians for >2 years, and whole-county HPSA designation). Race and ethnicity were collected as part of the birth certificate information for the purposes of public health tracking, health disparityies monitoring, and resource allocation.

Analyses were conducted between January 10 and June 28, 2025. We used R, version 4.0.3 (R Project for Statistical Computing).

## Results

Our sample included 13 765 184 live births (aged <20 [8.1%], 20-35 [78.0%], or >35 [13.8%] years; Black [13.9%], Hispanic [15.5%], or White [62.2%]) across 1381 primary care HPSA counties without any NHSC maternity care clinicians before the 2009 NHSC expansion. Of these counties, 190 (13.8%) received NHSC maternity care clinicians since 2009, of which 96 (50.5%) had high SVI.

**Figure 1.**
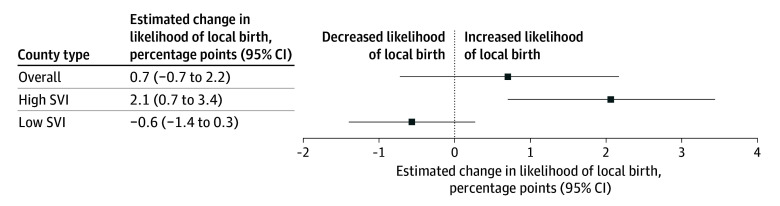
US Counties Receiving National Health Service Corps Maternal Care Clinicians and Probability of Local Childbirth, 2005 to 2019 Each box represents the change in the proportion of births that took place within the mother’s county of residence in treated counties relative to control counties from before to after the 2009 National Health Service Corps expansion. Error bars represent 95% CIs. Each difference-in-differences estimate accounts for maternal demographic characteristics, maternal health risks and comorbidities, birthing characteristics, non–National Health Service Corps maternity care clinician density (per 1000 females of reproductive age), hospital obstetric unit closure, state Medicaid expansion, year-month fixed effects, and county fixed effects. SVI indicates Social Vulnerability Index.

**Figure 2.**
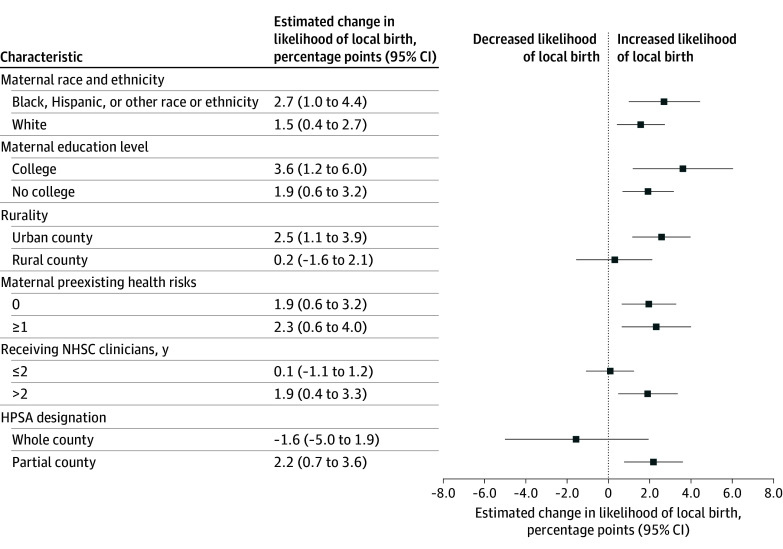
US Counties Receiving National Health Service Corps (NHSC) Maternal Care Clinicians and Probability of Local Childbirth in High Social Vulnerability Index Counties, by Maternal and County Characteristics, 2005 to 2019 Each box represents the change in the proportion of births that took place within the mother’s county of residence in treated counties relative to control counties from before to after the 2009 NHSC expansion. Error bars represent 95% CIs. Each difference-in-differences estimate accounts for maternal demographic characteristics, maternal health risks and comorbidities, birthing characteristics, non-NHSC maternity care clinician density (per 1000 reproductive-age females), hospital obstetric unit closure, state Medicaid expansion, year-month fixed effects, and county fixed effects. HPSA indicates Health Professional Shortage Area.

In high-SVI counties, NHSC expansion was associated with increased (2.1 [95% CI, 0.7-3.4] percentage points) likelihood of local birth (relative to the 72.0% baseline; Figure 1), corresponding to 11 346 additional local births. This increase was most pronounced in urban counties (2.5 [95% CI, 1.1-3.9] percentage points), counties with partial county shortage designations (2.1 [95% CI, 0.7-3.6] percentage points), and counties receiving NHSC clinicians for more than 2 years (1.8 [95% CI, 0.4-3.3] percentage points) (Figure 2). The increase was consistent across maternal race and ethnicity, education level, and preexisting health risks. However, we observed no significantly improved likelihood of local birth in low SVI counties or overall.

## Discussion

Our findings suggest that the 2009 NHSC expansion was associated with significantly increased local childbirths in high-SVI areas, benefiting diverse groups of birthing individuals. These findings align with prior research^6^ and strengthen evidence supporting workforce expansion as a strategy to improve maternal care access. However, rural areas and whole-county shortage areas remained especially vulnerable.

Study limitations include birth certificate data not identifying births attended by NHSC clinicians and lacking geographic details to calculate within-county travel distance. Our findings have implications for implementing the 2018 Improving Access to Maternity Care Act, which also aims to deploy NHSC clinicians to address health care access gaps in shortage areas. By identifying where past NHSC investments yielded measurable gains and where communities remained vulnerable, our study highlights key areas for prioritizing NHSC maternity care clinicians.

## Notes

Supplement 1. **eAppendix.** Background
eMethods

eReferences

Supplement 2.
Data Sharing Statement

## References

[1] Meyer E, Hennink M, Rochat R, . Working towards safe motherhood: delays and barriers to prenatal care for women in rural and peri-urban areas of Georgia. Matern Child Health J. 2016;20(7):1358-1365. doi:10.1007/s10995-016-1997-x 27053128

[2] Nowhere to go: maternity care deserts across the U.S. March of Dimes. 2020. Accessed August 15, 2023. https://www.marchofdimes.org/sites/default/files/2022-10/2020-Maternity-Care-Report.pdf

[3] Rayburn WF, Klagholz JC, Murray-Krezan C, Dowell LE, Strunk AL. Distribution of American Congress of Obstetricians and Gynecologists fellows and junior fellows in practice in the United States. Obstet Gynecol. 2012;119(5):1017-1022. doi:10.1097/AOG.0b013e31824cfe50 22525913

[4] Heisler EJ. The National Health Service Corps. Congressional Research Service. 2022. Accessed November 10, 2024. https://www.congress.gov/crs-products/product/pdf/R/R44970/15

[5] Pathman DE, Konrad TR. Growth and changes in the National Health Service Corps (NHSC) workforce with the American Recovery and Reinvestment Act. J Am Board Fam Med. 2012;25(5):723-733. doi:10.3122/jabfm.2012.05.110261 22956708

[6] Ma Y, Baker O, Zhang F, Cochran-McClain C, Kaimal AJ, Yu H. Prenatal care utilization and birth outcomes after expansion of the National Health Service Corps. Obstet Gynecol. 2024;144(4):526-535. doi:10.1097/AOG.0000000000005704 39173176 PMC11499018

