# Supplementary material for: Maternal Health Workforce Expansion and Local Childbirths
Source: JAMA Netw Open. 2026 Feb 2;9(2):e2556775. doi: 10.1001/jamanetworkopen.2025.56775 (PMC12865653; doi:10.1001/jamanetworkopen.2025.56775)
Supplement: Supplement 1. — eAppendix. Background eMethods eReferences [file jamanetwopen-e2556775-s001.pdf]

# Supplemental Online Content

Ma Y, Baker O, Zhang F, Cochran-McClain C, Kaimal A, Yu H. Maternal health workforce expansion and local childbirths. *JAMA Netw Open*. 2026;9(2):e2556775. doi:10.1001/jamanetworkopen.2025.56775

**eAppendix.** Background

**eMethods**

**eReferences**

This supplemental material has been provided by the authors to give readers additional information about their work.

© 2026 Ma Y et al. *JAMA Network Open*.

**eAppendix.** Background

The National Health Service Corps (NHSC) was created by the Emergency Health Personnel Act of 1970 to increase the supply of trained health providers in federally designated HPSAs.[1] It is the largest public program that provides scholarships and loan repayments to clinicians in exchange for their service in Health Professional Shortage Areas (HPSAs) designated by the Health Resources and Services Administration (HRSA).[2,3] NHSC maternity care clinicians, deemed part of the primary care workforce by HRSA, have been serving in counties with primary care HPSA designations.

The NHSC was substantially expanded in 2009 under the American Recovery and Reinvestment Act (ARRA),[4] a stimulus package enacted by Congress and signed into law by President Barack Obama in February 2009 in response to the 2008 financial crisis. The ARRA designated $300 million to expand the NHSC, more than doubled its annual budget. The NHSC expansion continued under the Affordable Care Act of 2010, which permanently reauthorized the NHSC program and created a new mandatory funding stream for the it.[2] As a result, the NHSC primary care workforce (including maternity care clinicians) increased by 136% between 2009 and 2011,[3,5] and more than tripled by 2022.[2,3]

© 2026 Ma Y et al. *JAMA Network Open*.

## eMethods

1. **Data**

   We consolidated data from multiple sources to conduct the analyses, including: (1) 2005-2019 birth certificate data from the CDC's National Center for Health Statistics; (2) county-level annual counts of NHSC maternity care clinicians (including ob-gyns, family physicians providing maternity care, certified nurse-midwives, nurse practitioners specializing in women's health, and physician assistants specializing in women's health) from HRSA; (3) county-level annual primary care HPSA designations from HRSA's Area Health Resources Files; (4) county-level annual total counts of maternity care clinicians, including counts of ob-gyns and family physicians from the Area Health Resources Files, and counts of non-physician maternity care clinicians (i.e., certified nurse midwives, certified midwives, and physician assistants and nurse practitioners providing obstetrics-gynecology services) from the National Plan and Provider Enumeration System by the Centers for Medicare and Medicaid Services (CMS);[6] (5) annual county-level availability of hospital-based obstetric unit availability from American Hospital Association (AHA) Annual Survey of Hospitals data and Centers for Medicare & Medicaid Services (CMS) Provider of Services (POS) File;[7,8] and (6) state-level information about the ACA Medicaid expansion from the Kaiser Family Foundation website.[9] To build the analytic data file, we linked the birth certificate data with the above state and county-level data sources using year and state/county identifier.

2. **Treated vs Control Counties**

   We first identified counties that were designated as primary care HPSAs every year between 2005 and 2019.[10,11] Counties with either whole-county or partial-county HPSA designations were included in the analysis, because both were eligible for receiving NHSC clinicians.[12,13] Among these shortage counties, we then identified a subset of counties that did not have any NHSC maternity care clinicians prior to the 2009 NHSC expansion. Among these counties, those that received NHSC maternity care clinicians for at least one year after the 2009 NHSC expansion were classified as treated counties, and those remained without any NHSC primary care clinicians after 2009 were classified as control counties.

3. **Study Sample**

   We limited our study sample to live births taking place in treated and control counties between 2005 and 2019. Our study period began in 2005 to allow consistent coding of key variables under the 2003 birth certificate revision across all states. Before 2005, some states used the 2003 version while other states used the 1989 version. Our study period ended in 2019 to avoid the known COVID-19-related disruptions to birthing patterns and delivery choices.[14]

© 2026 Ma Y et al. *JAMA Network Open*.

4. **Outcome Variables**
   Among live births in our study counties during the study period, we examined whether the birth took place in the same county as the mother's residence.

5. **Covariates**
   Our study accounted for individual-, county-, and state-level factors that may affect the likelihood of local childbirth. Individual-level covariates included maternal demographic characteristics (including age, race and ethnicity, marital status, and education), maternal health risks and comorbidities (including tobacco smoking status, diabetes, chronic hypertension, and gestational hypertension), birthing characteristics (including birthing facility [in hospital versus not in hospital], plurality [singleton versus non-singleton birth], and birth order [first-time birth versus non-first-time birth]). County-level covariates included annual densities of non-NHSC maternity care clinicians (defined as the difference between the total number of maternity care clinicians and the number of NHSC maternity care clinicians per 1,000 reproductive age women). Specifically, we included both density of non-NHSC maternity care physicians (i.e., ob-gyns and family physicians providing maternity care services [15–17]) and density of other non-NHSC maternity care clinicians (certificated nurse midwives, certificated midwives, and midwives). County-level covariates also included whether a county lost all in-county hospital-based obstetric services in a given year. State-level covariates included the ACA Medicaid expansion.

6. **Study Design**
   We used a generalized difference-in-differences design with county and year-month two-way fixed effects to compare changes in likelihood of local childbirth between birthing people residing in treated and control counties from before to after the NHSC expansion.[18,19] The average treatment effect on the treated counties was captured through a binary indicator denoting the presence of any NHSC maternity care clinicians in a county for a given year. The estimated coefficient for this binary indicator represents the average change in probability of local birth for birthing people residing in the treated counties relative to control counties after the NHSC expansion. Since NHSC clinicians typically serve two years under their initial contracts and then may either extend or terminate their contracts,[20] such design allowed us to account for the fact that some counties that initially received NHSC maternity care clinicians through the NHSC expansion may subsequently lose their NHSC clinicians, while other counties continued to have NHSC maternity care clinicians.[18,19]

   We estimated a logistic model for the likelihood of local childbirth, i.e., having childbirth in the same county as the mother's residence. The dependent variable is the binary indicator for local childbirth, and the key explanatory variable is a binary indicator denoting the presence of any NHSC maternity care clinicians in a county for a given year, adjusting for maternal demographic characteristics, maternal health risks and comorbidities, birthing characteristics,

© 2026 Ma Y et al. *JAMA Network Open*.

non-NHSC maternity care clinician density, county hospital obstetric unit closure, state Medicaid expansion, year and month fixed effects, and county fixed effects. Standard errors were clustered at state level to account for any potential correlation within state.

To understand whether the NHSC expansion since 2009 had differential effects on neighborhoods of different social vulnerability, we stratified our analyses by birthing person's residence in high versus low social vulnerability areas as classified by the CDC.[21] We also stratified the analysis by birthing person's race and ethnicity (White vs Non-White), educational attainment (college vs no college), rurality (urban vs rural), health risk status (with diabetes/hypertension vs no diabetes/hypertension), the duration of NHSC clinician presence in the county (two years or less vs more than two years), and the county's type of HPSA shortage designation (whole-county vs partial-county shortage). Rurality was defined using the 2013 National Center for Health Statistics (NCHS) Urban–Rural Classification Scheme for Counties, with rural counties classified as those assigned codes 5 or 6 (micropolitan or noncore).

To assess the validity of the difference-in-differences design, we tested for difference in pre-NHSC expansion linear trends using pre-NHSC expansion data (see the following table) and established that trends in the study outcome were similar between the treated and control counties for the main analysis and each of the subgroup analyses.

**Assessing Validity of Difference-in-Differences Design: Test for Difference in Linear Time Trend in the Pre Period**

| | Estimate | Std. Error | P Value |
|---|---|---|---|
| Overall | -0.013 | 0.017 | 0.445 |
| | | | |
| High Social Vulnerability Counties | -0.001 | 0.012 | 0.942 |
| Low Social Vulnerability Counties | -0.007 | 0.010 | 0.484 |
| | | | |
| Among High Social Vulnerability Counties | | | |
| White | -0.019 | 0.032 | 0.556 |
| Non-White | 0.015 | 0.028 | 0.590 |
| | | | |
| College | -0.038 | 0.048 | 0.428 |
| No College | 0.005 | 0.023 | 0.845 |
| | | | |
| Urban | 0.001 | 0.034 | 0.979 |
| Rural | 0.015 | 0.020 | 0.448 |
| | | | |
| No Health Risk | -0.004 | 0.024 | 0.883 |
| With Health Risk | -0.011 | 0.053 | 0.833 |
| | | | |
| Receiving NHSC Clinicians ≤2 Years | -0.073 | 0.076 | 0.337 |
| Receiving NHSC Clinicians >2 Years | 0.018 | 0.018 | 0.319 |

© 2026 Ma Y et al. *JAMA Network Open*.

| | | | |
|---|---|---|---|
| Whole-County Shortage | -0.001 | 0.063 | 0.983 |
| Partial-County Shortage | 0.013 | 0.020 | 0.518 |

Notes:

1. To assess whether there is any difference in outcomes between the treated counties and control counties prior to the 2009 NHSC expansion, each outcome is regressed on a binary indicator for treatment counties, a linear year term, an interaction term between treatment counties and the linear year term, maternal demographic characteristics, maternal health risks and comorbidities, birthing characteristics, non-NHSC maternity care clinician density, hospital obstetric units closure, state Medicaid expansion, year and month fixed effects, and county fixed effects, using the 2007-2009 birth data only (i.e., prior to the 2009 NHSC expansion took effect).
2. "Estimate" represents the coefficient of the interaction term between treatment counties and the linear year variable. We found that the coefficient for each interaction term is not statistically significant, suggesting that there is no significant difference in the trends of the study outcome between treated and control counties prior to the 2009 NHSC expansion.

© 2026 Ma Y et al. *JAMA Network Open*.

## eReferences


1. Heisler EJ. The National Health Service Corps. Congress.gov. April 9, 2025. Accessed November 18, 2025. https://www.congress.gov/crs-product/R44970

2. Heisler EJ. *The National Health Service Corps*. Congressional Research Service; 2022. Accessed November 10, 2024. https://crsreports.congress.gov/product/pdf/R/R44970/15

3. Health Resources & Services Administration. *National Health Service Corps Report to Congress for The Year 2020*. Health Resources & Services Administration; 2020. https://bhw.hrsa.gov/sites/default/files/bureau-health-workforce/about-us/reports-to-congress/nhsc-report-congress-2020.pdf

4. U.S. Department of Health and Human Services. *A 21st Century Health Care Workforce for the Nation*. U.S. Department of Health and Human Services; 2014. https://aspe.hhs.gov/sites/default/files/private/pdf/76796/rpt_healthcareworkforce.pdf

5. Pathman DE, Konrad TR. Growth and changes in the National Health Service Corps (NHSC) workforce with the American Recovery and Reinvestment Act. *J Am Board Fam Med JABFM*. 2012;25(5):723-733. doi:10.3122/jabfm.2012.05.110261

6. Vanderlaan J, Jefferson K. Evaluation of a method to identify midwives in national provider identifier data. *BMC Pregnancy Childbirth*. 2023;23(1):809. doi:10.1186/s12884-023-06122-2

7. Kozhimannil KB, Hung P, Henning-Smith C, Casey MM, Prasad S. Association Between Loss of Hospital-Based Obstetric Services and Birth Outcomes in Rural Counties in the United States. *JAMA*. 2018;319(12):1239-1247. doi:10.1001/jama.2018.1830

8. Interrante JD, Carroll C, Handley SC, Kozhimannil K. An Enhanced Method for Identifying Hospital-Based Obstetric Unit Status. University of Minnesota Rural Health Research Center. January 2022. Accessed May 30, 2025. https://rhrc.umn.edu/wp-content/uploads/2022/01/UMN-OB-Unit-Identification-Methods_7.pdf

9. Status of State Action on the Medicaid Expansion Decision. Kaiser Family Foundation. https://www.kff.org/health-reform/state-indicator/state-activity-around-expanding-medicaid-under-the-affordable-care-act/?currentTimeframe=0&sortModel=%7B%22colId%22:%22Location%22,%22sort%22:%22asc%22%7D

10. Health Resources & Services Administration. Scoring Shortage Designations. HRSA Health Workforce. December 2022. Accessed June 17, 2024. https://bhw.hrsa.gov/workforce-shortage-areas/shortage-designation/scoring

11. The authors have previously verified with the Health Resources & Services Administration that the HPSA designation of a county would not be changed after receiving NHSC clinicians. Source: Email exchanges between Dr. Hao Yu and Ms. Kourtney Thomas and Ms. Michelle Goodman of the Health Resources & Services Administration on April 4, 2016.


© 2026 Ma Y et al. *JAMA Network Open*.

12. Health Resources & Services Administration. What Is Shortage Designation. HRSA Health Workforce. Accessed November 18, 2025. https://bhw.hrsa.gov/workforce-shortage-areas/shortage-designation#hpsas

13. Health Resources & Services Administration. How to Meet NHSC Site Eligibility Requirements. HRSA National Health Service Corps. May 2025. Accessed November 18, 2025. https://nhsc.hrsa.gov/sites/eligibility-requirements

14. Silverstein H, Jennings Mayo-Wilson L, Austin A. Changes in the utilization of prenatal and delivery services in the United States prior to, during, and after the COVID-19 pandemic. *Reprod Health*. 2025;22(1):136. doi:10.1186/s12978-025-02084-0

15. Number of general practitioners and family physicians providing obstetrics-gynecology services are derived by multiplying annual total number of general practitioners and family physicians in the Area Health Resources Files with the proportion of family physicians providing maternity care services documented in the literature.

16. Tong ST, Makaroff LA, Xierali IM, Puffer JC, Newton WP, Bazemore AW. Family physicians in the maternity care workforce: factors influencing declining trends. *Matern Child Health J*. 2013;17(9):1576-1581. doi:10.1007/s10995-012-1159-8

17. Sebastian T. Tong, Zachary J. Morgan, Andrew W. Bazemore, Aimee R. Eden, Lars E. Peterson. Maternity Access in Rural America: The Role of Family Physicians in Providing Access to Cesarean Sections. *J Am Board Fam Med*. 2023;36(4):565. doi:10.3122/jabfm.2023.230020R1

18. Goodman-Bacon A. Difference-in-differences with variation in treatment timing. *J Econom*. 2021;225(2):254-277. doi:10.1016/j.jeconom.2021.03.014

19. Kessler DP, Sage WM, Becker DJ. Impact of Malpractice Reforms on the Supply of Physician Services. *JAMA*. 2005;293(21):2618-2625. doi:10.1001/jama.293.21.2618

20. Health Resources & Services Administration. Determine Your Eligibility and Apply for a Continuation Contract. Accessed October 4, 2023. https://nhsc.hrsa.gov/loan-repayment/continuation-contract

21. CDC/ATSDR Social Vulnerability Index. Agency for Toxic Substances and Disease Registry. Accessed September 28, 2023. https://www.atsdr.cdc.gov/placeandhealth/svi/index.html

© 2026 Ma Y et al. *JAMA Network Open*.
