# Supplementary material for: Maternal Health Workforce Expansion and Local Childbirths
Source: JAMA Netw Open. 2026 Feb 2;9(2):e2556775. doi: 10.1001/jamanetworkopen.2025.56775 (PMC12865653; doi:10.1001/jamanetworkopen.2025.56775)
Supplement: Supplement 2. — Data Sharing Statement [file jamanetwopen-e2556775-s002.pdf]

Data Sharing Statement

Ma. Maternal Health Workforce Expansion and Local Childbirths. *JAMA Netw Open*. Published February 02, 2026. doi:10.1001/jamanetworkopen.2025.56775

Data
Data available: No
